# Evidence Review and Practice Recommendation on the Material, Design, and Maintenance of Cloth Masks

**DOI:** 10.1017/dmp.2020.317

**Published:** 2020-09-02

**Authors:** Anthony Paulo Sunjaya, Lidia Morawska

**Affiliations:** Respiratory Division, The George Institute for Global Health, UNSW Sydney, Australia; International Laboratory for Air Quality and Health (ILAQH), School of Earth and Atmospheric Sciences, Queensland University of Technology, Brisbane, Queensland, Australia

**Keywords:** coronavirus disease 2019, face masking, homemade masks, viral infection

## Abstract

Despite numerous masking recommendations from public health agencies, including the World Health Organization, editorials, and commentaries providing support for this notion, none had examined different homemade masks or demonstrated that perhaps not all cloth masks are the same. This article aims to provide evidence-based recommendations on cloth-mask materials, its design, and, importantly, its maintenance. Articles were obtained from PubMed and preprint servers up to June 10, 2020. Current evidence suggests that filtration effectiveness can range from 3% to 95%. Multiple layer (hybrid) homemade masks made from a combination of high density 100% cotton and materials with electrostatic charge would be more effective than one made from a single material. Mask fit greatly affects filtration efficiency, and adding an overhead knot or nylon overlay potentially provides the best fit for cloth masks. There is a paucity of evidence for masks maintenance as most studies are in the laboratory setting; however, switching every 4 hours as in medical masks and stored in dedicated containers while awaiting disinfection is recommended. Outside of these recommendations to improve the effectiveness of cloth masks to reduce infection transmission, there is a need for countries to set up independent testing labs for homemade masks made based on locally available materials. This can use existing occupational health laboratories usually used for accrediting masks and respirators.

Worldwide, face masking has become a global phenomenon associated with the coronavirus disease (COVID-19) pandemic. This is even more important with countries removing lockdowns. Masking itself has been a common sight in many Asian countries who had faced the 2003 severe acute respiratory syndrome coronavirus (SARS-CoV) pandemic and several other recent epidemics even before the COVID-19 pandemic.^1,2^ During the pandemic, an online survey at the end of January 2020 in Hubei, China, reported that up to 98% of respondents were using masks outdoors.^3^

Recently, the World Health Organization and the US Centers for Disease Control and Prevention (CDC) have reversed their recommendation to advocate for universal masking with the public recommended to use homemade cloth masks and not medical masks.^4,5^ The use of cloth masks to prevent infection is nothing new. It had been used for decades in surgical theaters before being replaced by the more effective medical masks and are still in use in many low-resource settings.^6^ Prior to the current public masking recommendation, the CDC had provided recommendation for health professionals on the use of homemade masks (scarfs, bandana) as a last resort in settings where face masks are not available.^5^ Even so, despite numerous masking recommendations from public health agencies, editorials and commentaries providing support for this notion, none had examined the different homemade masks or demonstrated that perhaps not all cloth masks are the same.^7,8^

This is important because the materials used, how they are worn, and their maintenance play an important role in determining whether they’ll be effective in reducing risk of transmission or may potentially bring even harm to the wearer. This article aims to examine current evidence on cloth masks and, with theoretical rationales, provide some recommendations on what might be an effective cloth mask for the public.

## METHODS

Articles for the narrative review were searched from PubMed and preprint server (medRxiv) and last updated on June 10, 2020. We also hand searched references of previous reviews on masking and references of the included studies.

Search terms used include (masks OR face masks OR fabric masks OR cloth masks OR homemade masks) AND (infection OR SARS OR SARS-CoV-2 OR coronavirus OR influenza OR COVID-19).

Studies of any type were included. We only included studies that were published in full and in the English language.

### Cloth-Mask Materials

While a recent study has shown that surgical masks are effective in preventing transmission of human coronaviruses found in exhaled breath, there have been no head-to-head studies for surgical versus cloth/homemade masks’ effectiveness against severe acute respiratory syndrome coronavirus 2 (SARS-CoV-2), the causative virus of COVID-19 in the community.^9^ However, there have been laboratory experiments conducted to assess particle filtering effectiveness of cloth masks based on the percentage of particles penetrating the material (particle penetration) and a few studies examining the effectiveness of cloth masks against influenza and other coronaviruses.

A recent study by Ma et al. comparing the efficacy of N95 masks, medical masks, and homemade masks (made from 4 layers of kitchen paper and 1 layer cloth) against avian influenza to mock the coronavirus reported that 99.98%, 97.14%, and 95.15%, respectively, of the virus in aerosols made by a nebulizer were blocked.^10^ Another study by van der Sande similarly found that a homemade mask (made from tea cloth) decreases viral exposure and infection risk. Even so, it provides about half as much the protection (measured as an inverse of particle leakage) as surgical masks, which the authors suggest is due to its imperfect fit.^11^

The pillowcase and 100% cotton T-shirt have also been reported to be the most suitable material for an improvised face mask based on a study of 10 materials by Davies et al.^12^ They reported that, for influenza, homemade masks have one-third the effectiveness of medical masks, although homemade masks were able to significantly reduce the number of microorganisms expelled compared to no protection.^12^

However, a cluster randomized trial of cloth masks (2 layers made of cotton) compared with medical masks among health care workers conducted by MacIntyre et al. reported the particle penetration of cloth masks and medical masks as 97% and 44%, respectively. The study also reported that there was a significantly higher risk of influenza like illness (adjusted RR = 6.64, 95% CI: 1.45 to 28.65) and laboratory-confirmed virus (adjusted RR = 1.72, 95% CI: 1.01 to 2.94) in the cloth mask group compared to medical mask group.^6^

Not all cloth mask materials provide the same protection from particle penetration, which may potentially be 1 explanation to the differing findings. A laboratory test conducted among 44 different masks of 5 types – yellow sand, quarantine, medical, general masks, and handkerchiefs – showed great variability in particle penetration characteristics. An interesting finding with regard to general masks is the differing particle penetration between those made from nonwoven and cotton. Nonwoven masks resulted in significantly less particle penetration compared to cotton masks with mean particle penetration of 53% versus 70%, respectively, based on particle penetration measurements performed using the Korean Food and Drug Administration (similar to European Union test protocol) method. Furthermore, handkerchiefs made of cotton and gauze even up to 4 layers thick reported a particle penetration of 87% and 97%, respectively.^13^

Similarly, another laboratory test conducted by the US National Institute for Occupational Safety and Health laboratory reported that cloth masks and other fabrics tested against polydisperse and monodisperse NaCl aerosols (20–100 nm) showed 40% to 90% particle penetration, with the best material being a 100% cotton sweatshirt compared with a mix of cotton and polyester or 100% polyester.^14^ The absence of electrostatic charge in the fabric tested in the study may play a role in its high particle penetration. Interestingly, these particle penetration levels were reported to be similar to some commonly used US Food and Drug Administration approved surgical masks and unapproved dust masks that have particle penetration levels of 51% to 89% in similar testing. It must also be noted that previous studies have shown that even N95 masks, while able to filter 95% of particles greater than 3 nm in size, are marginally beneficial against particles < 2.5 µm in diameter as tested against in this study.

This was supported by the result of Shakya et al.’s study, which shows that the filtration effectiveness of cloth masks was better for particles larger than 1 µm in diameter (64–94%) compared with those smaller than 1 µm (44–93%).^15^ A feature they report to perform better is cloth masks with exhalation valves and conical in shape to fit the face, although it must be noted that this study aims to evaluate the effectiveness in blocking toxic fumes not biological particles.

A recent laboratory study also showed that higher density cotton (600 threads per inch) prevents penetration of particles < 300 nm by > 65% and penetration of particles > 300 nm in diameter by > 90%.^16^ Interestingly, the quilt, a common household item made of 90% cotton–5% polyester–5% other fibers was found to perform even better blocking penetration of > 80% for particles < 300 nm and > 90% for > 300 nm. Combining high density cotton with silk, 2 layers of chiffon (90% polyester, 10% spandex), and 1 layer of flannel (65% cotton, 35% polyester) also provided a similar effectiveness. Although they were all slightly inferior to N95 in filtering particles above 300 nm, they were superior for particles < 300 nm.^16^

There are several key insights that can be gained from the above studies on mask materials, including the following:

1. Nonwoven materials and, in some cases, high density 100% cotton seem to provide the best particle filtration capacity, especially for larger-sized particles.
2. Materials with electrostatic charge (such as silk, polyester, and other synthetic fibers) are superior to the above materials for filtering smaller-sized particles (< 300 nm).
3. There’s limited evidence that simply adding the number of layers from the same material may not provide a significant increase in effectiveness.

Hence, it can be argued as 1 study has shown that a multiple layer (hybrid) homemade mask made from a combination of high density 100% cotton and materials with electrostatic charge would be more effective than a mask made from a single material. Prolonged use of masks is not always suitable for everyone, breathability considerations also come into play when preparing one’s own mask, and those with chronic lung disease, such as chronic obstructive pulmonary disease, may consider using a mask with fewer layers to improve breathability and adherence to use in daily life. Furthermore, for the studies against influenza, we must note that, while both SARS-CoV-2 and influenza are respiratory viruses, they belong to different families and hence have different transmissibility, which will limit the generalizability of those findings to the current pandemic. Even so, they provide some signal of benefit in reducing penetration of viral particles.

### Mask Design

**FIGURE 1:**
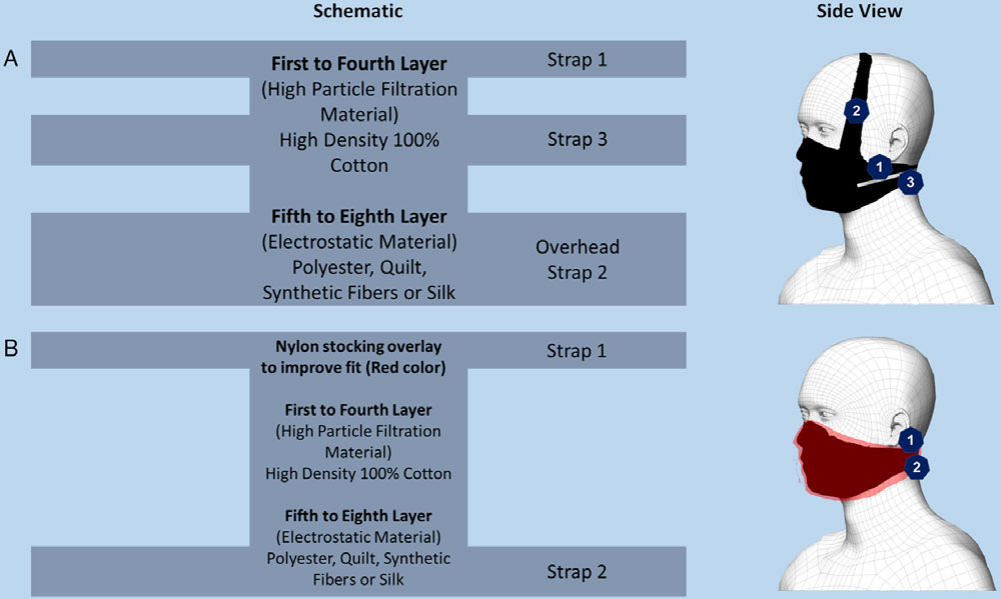
Schematics and Side View of Mask A (with overhead strap) and Mask B (with nylon overlay) Based on the Previous Findings. Both the overhead strap and nylon overlap aim to improve fit. Straps are numbered based on the order of tie.

Respirators, such as N95 and FFP masks, are protective not only due to their particle filtering capabilities, but also due to their fit, unlike surgical masks and cloth masks that do not fit tightly. A recent study has showed that mask filtration efficiency can fall by up to 60% when improperly fitted.^16^ With this regard, design factors can play a role in improving fit as shown in the study by Dato et al., which showed that a fit factor of 67 (N95’s fit factor is 100) can be achieved with cloth masks.^17^ Here, a cloth masks were made with 3 knot points – 2 behind the head and 1 over the head with 8 inner layers in the center piece.^17^ A recent study by Mueller et al.^18^ also showed the potential to improve fit through the use of a nylon stocking layer over the masks to cover all the edges. This reduces the flow of air around the edges and was reported to improve the particle filtration efficiency by 15% to 50%. Find below the schematic for a mask design that we recommend based on these studies (Figure 1).

Cloth masks during the pandemic have the main goal of source control, reducing transmission of droplets containing viral particles. Even so, these particles remain larger than the ultrafine particles (< 100 nm in size) from air pollution or toxic fumes.^1^ Hence, it can be argued that a perfect fit is not the main aim of cloth masks as that itself is not a feature of medical masks. However, a better fit and less leakage will provide greater potential to reduce aerosol penetration; simple adjustments to the design of homemade masks and adding a nylon stocking layer can aid in this. This is especially more important in light of recent evidence suggesting airborne transmission of SARS-CoV-2, especially in enclosed spaces.^19^ Importantly, the public must be made to understand the importance of a mask covering until the bridge of one’s nose and chin, not having it hanging and only covering the mouth.

### Mask Maintenance

There is a paucity of evidence with regard to mask maintenance. Most studies reported previously are conducted in the laboratory setting where their use is limited to 1 to 2 hours and only once. The only community study conducted in Vietnam^6^ in health care workers compared the use of cloth masks (1 per day) with another arm wearing medical masks replaced every 4 hours. This study design may be 1 reason to explain the differing results in this study and the short laboratory experiments. A previous study has shown that a longer duration of mask use (> 6 hours) resulted in a significantly higher percentage of masks containing viral particles.^20^ In such a scenario, the mask becomes a fomite and not prevent but may transmit infection.

Reflecting on this, it is our view that one should have multiple masks and that cloth masks should be worn and changed for much shorter periods of time or at least the same with medical mask guidelines, which is ideally a mask every 4 hours or until damp. Considering that this will be used by the community, an easy reminder to change masks is to have a mask change during the mealtime when it must be taken off. This would help promote adherence to this recommendation. As what we are discussing here is about community use, not in health care workers, people will not have the discipline to wear masks properly for long periods of time. It can be argued that they may not have to. There is rationale to recommend its wear only in public, especially during commuting to an office where they can have it removed as long as they are physically distanced from others and have sufficient ventilation, preventing a buildup of virus-laden droplets in the air.

One other critical aspect is what happens with the mask after the user takes it off, say, after lunch, when they are ready for the second mask for the day? A surgical mask would be thrown away at this stage as they are recommended for use for only 4 hours. However, this cloth mask will not. Therefore, together with a mask, people will need to have a container where they would store the contaminated mask before they can wash them. This is very practical, maybe too obvious, but we suspect that many people will not worry about this and put them into the pocket, handbag, keep it hanging on their neck, and so on, carrying with them a reservoir of the virus. Plastic containers or Ziploc bags, which are disposable or easy to disinfect, would be possible options for storage.

Being reusable, cleaning the mask after use is another important aspect that should not be taken for granted. How should these masks be best cleaned? Alcohol, while an effective disinfectant, has been shown in previous studies to reduce effectiveness of masks. A study by Martin Jr and Moyer et al. showed that cleaning respirators (N95, N99, R95, P100) by dipping them into isopropanol (alcohol rub) for 15 seconds and then dried resulted in a substantial increase in penetration to up to 65%.^21^ This is a result of isopropanol reducing or eliminating any electrostatic charge on the fibers, which, discussed previously, has filtering properties.

While there have been no studies examining different cleaning mechanisms and eradication of viral particles, the CDC recommends washing using hot water 70–80°C for 10 minutes using detergent.^22^ Even so, other studies have shown that temperatures above 50°C are significantly able to reduce microorganisms even without the use of detergents.^23^ These temperatures are the rationale to follow in cleaning homemade masks, based on reports during SARS-CoV that they are eradicated at temperatures above 56°C for 15 minutes or when exposed to humidity above 95%. Hence, exposure to hot water for 15 minutes can also be a readily accessible way to clean the masks.^24^

Outside of washing, drying with the sun’s ultraviolet (UV) exposure has been proposed as a way to disinfect. However, while previous studies have shown that UV rays helped inactivate SARS-CoV, it was only UVC that provided the required germicidal properties, which is not present from sun rays as they’re filtered by the atmosphere.^25^

## FUTURE IMPLICATIONS

Outside of these recommendations, to improve the effectiveness of cloth masks to reduce infection transmission, there is a need for countries to set up independent testing labs for homemade masks made based on locally available materials. This can use existing occupational health laboratories usually used for accrediting medical masks and respirators.

The COVID-19 pandemic is a time to push for innovation in the development of personal protective equipment for health care workers and the community. While recommending that universal masking is something to support, the use of masks, which may previously appear mundane and low tech, requires proper design, wear, and maintenance for it to be effective enough to support reducing transmission during the pandemic.

## References

[1] Huang W , Morawska L. Face masks could raise pollution risks. Nature. 2019;574(7776):29-30. 31576041 10.1038/d41586-019-02938-1

[2] Elachola H , Ebrahim SH , Gozzer E. COVID-19: facemask use prevalence in international airports in Asia, Europe and the Americas, March 2020. Travel Med Infect Dis. 2020;35. doi: 10.1016/j.tmaid.2020.101637. PMC7118530 32205271

[3] Zhong BL , Luo W , Li HM , et al. Knowledge, attitudes, and practices towards COVID-19 among Chinese residents during the rapid rise period of the COVID-19 outbreak: a quick online cross-sectional survey. Int J Biol Sci. 2020;16(10):1745-1752. 32226294 10.7150/ijbs.45221 PMC7098034

[4] World Health Organization. Coronavirus disease (COVID-19) advice for the public: when and how to use masks. 2020 https://www.who.int/emergencies/diseases/novel-coronavirus-2019/advice-for-public/when-and-how-to-use-masks. Accessed June 10, 2020.

[5] Centers for Disease Control. Coronavirus disease 2019 (COVID-19) – facemasks. 2020 https://www.cdc.gov/coronavirus/2019-ncov/hcp/ppe-strategy/face-masks.html. Accessed May 9, 2020.

[6] MacIntyre CR , Seale H , Dung TC , et al. A cluster randomised trial of cloth masks compared with medical masks in healthcare workers. BMJ Open. 2015;5(4):e006577. 10.1136/bmjopen-2014-006577 PMC4420971 25903751

[7] Greenhalgh T , Schmid MB , Czypionka T , et al. Face masks for the public during the COVID-19 crisis. BMJ. 2020;369:m1435. 32273267 10.1136/bmj.m1435

[8] Sunjaya AP , Jenkins C. Rationale for universal face masks in public against COVID-19. Respirology. 2020. 10.1111/resp.13834 PMC7267357 32353901

[9] Leung NHL , Chu DKW , Shiu EYC , et al. Respiratory virus shedding in exhaled breath and efficacy of face masks. Nat Med. 2020;26(5):676-680. 32371934 10.1038/s41591-020-0843-2 PMC8238571

[10] Ma QX , Shan H , Zhang HL , et al. Potential utilities of mask-wearing and instant hand hygiene for fighting SARS-CoV-2. J Med Virol. 2020. 10.1002/jmv.25805 PMC7228401 32232986

[11] van der Sande M , Teunis P , Sabel R. Professional and home-made face masks reduce exposure to respiratory infections among the general population. PLoS One. 2008;3(7):e2618. 18612429 10.1371/journal.pone.0002618 PMC2440799

[12] Davies A , Thompson KA , Giri K , et al. Testing the efficacy of homemade masks: would they protect in an influenza pandemic? Disaster Med Public Health Prep. 2013;7(4):413-418. 24229526 10.1017/dmp.2013.43 PMC7108646

[13] Jung H , Kim JK , Lee S , et al. Comparison of filtration efficiency and pressure drop in anti-yellow sand masks, quarantine masks, medical masks, general masks, and handkerchiefs. Aerosol Air Qual Res. 2014;14(3):991-1002.

[14] Rengasamy S , Eimer B , Shaffer RE. Simple respiratory protection – evaluation of the filtration performance of cloth masks and common fabric materials against 20-1000 nm size particles. Ann Occup Hyg. 2010;54(7):789-798. 20584862 10.1093/annhyg/meq044 PMC7314261

[15] Shakya KM , Noyes A , Kallin R , et al. Evaluating the efficacy of cloth facemasks in reducing particulate matter exposure. J Expo Sci Environ Epidemiol. 2017;27(3):352-357. 27531371 10.1038/jes.2016.42

[16] Konda A , Prakash A , Moss GA , et al. Aerosol filtration efficiency of common fabrics used in respiratory cloth masks. ACS Nano. 2020. 10.1021/acsnano.0c03252 32329337

[17] Dato VM , Hostler D , Hahn ME. Simple respiratory mask. Emerg Infect Dis. 2006;12(6):1033-1034. 16752475 10.3201/eid1206.051468 PMC3373043

[18] Mueller AV , Eden MJ , Oakes JJ , et al. Assessment of fabric masks as alternatives to standard surgical masks in terms of particle filtration efficiency. medRxiv. 2020. 10.1016/j.matt.2020.07.006 PMC7346791 32838296

[19] Morawska L , Milton DK. It is time to address airborne transmission of COVID-19. Clin Infect Dis. 2020. 10.1093/cid/ciaa939 PMC7454469 32628269

[20] Chughtai AA , Stelzer-Braid S , Rawlinson W , et al. Contamination by respiratory viruses on outer surface of medical masks used by hospital healthcare workers. BMC Infect Dis. 2019;19(1). doi: 10.1186/s12879-019-4109-x. PMC6547584 31159777

[21] Martin SB Jr , Moyer ES. Electrostatic respirator filter media: filter efficiency and most penetrating particle size effects. Appl Occup Environ Hyg. 2000;15(8):609-617. 10957816 10.1080/10473220050075617

[22] Centers for Disease Control and Prevention. Healthcare-associated infections: linen and laundry management. 2020 https://www.cdc.gov/hai/prevent/resource-limited/laundryhtml. Accessed June 10, 2020.

[23] Bockmühl DP , Schages J , Rehberg L. Laundry and textile hygiene in healthcare and beyond. Microbial Cell. 2019;6(7):299-306. 31294042 10.15698/mic2019.07.682 PMC6600116

[24] Chan KH , Peiris JSM , Lam SY , et al. The effects of temperature and relative humidity on the viability of the SARS coronavirus. Adv Virol. 2011;2011:1-7. 10.1155/2011/734690 PMC3265313 22312351

[25] Darnell MER , Subbarao K , Feinstone SM , et al. Inactivation of the coronavirus that induces severe acute respiratory syndrome, SARS-CoV. J Virol Methods. 2004;121(1):85-91. 15350737 10.1016/j.jviromet.2004.06.006 PMC7112912

